# Supplementary material for: A CMOS silicon spin qubit
Source: Nat Commun. 2016 Nov 24;7:13575. doi: 10.1038/ncomms13575 (PMC5123048; doi:10.1038/ncomms13575)
Supplement: Supplementary Information — Supplementary Figures 1-8, Supplementary Notes 1-6 and Supplementary References [file ncomms13575-s1.pdf]

“A CMOS silicon spin qubit”: Supplementary information

Supplementary Figures

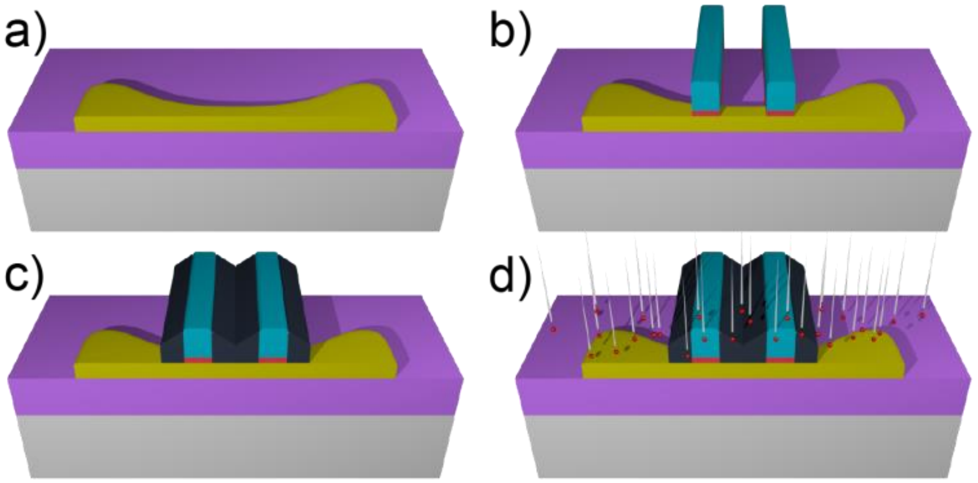

*Supplementary Figure 1 :**Device fabrication** a-d) Schematic illustration of some key steps in device fabrication : a) Silicon nanowire etching, b) Gate definition, c) Self-aligned insulating spacers around the gates, d) Self-aligned doping.*

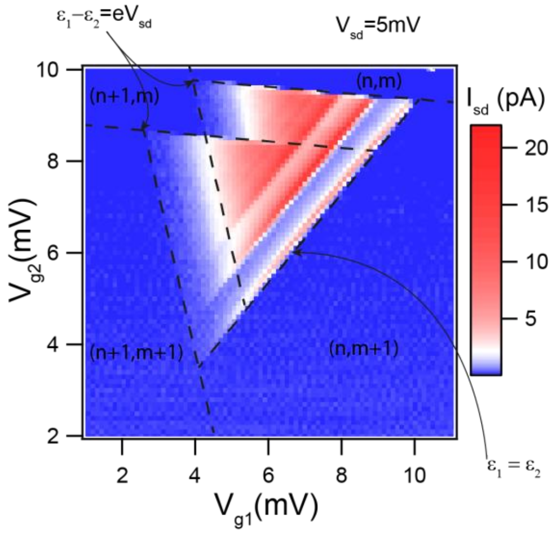

*Supplementary Figure 2: **Double dot bias triangles**: Source-drain current I_{sd} as a function of gate voltages V_{g1} and V_{g2} at $T = 10$ mK and $V_{sd} = 5$ mV. The observed current triangles result from the tunneling of holes through the double quantum dot, which is energetically allowed for $\mu1 \geq \varepsilon1 \geq \varepsilon2 \geq \mu2$. Integers n and m denote number of holes in QD1 and QD2, respectively.*

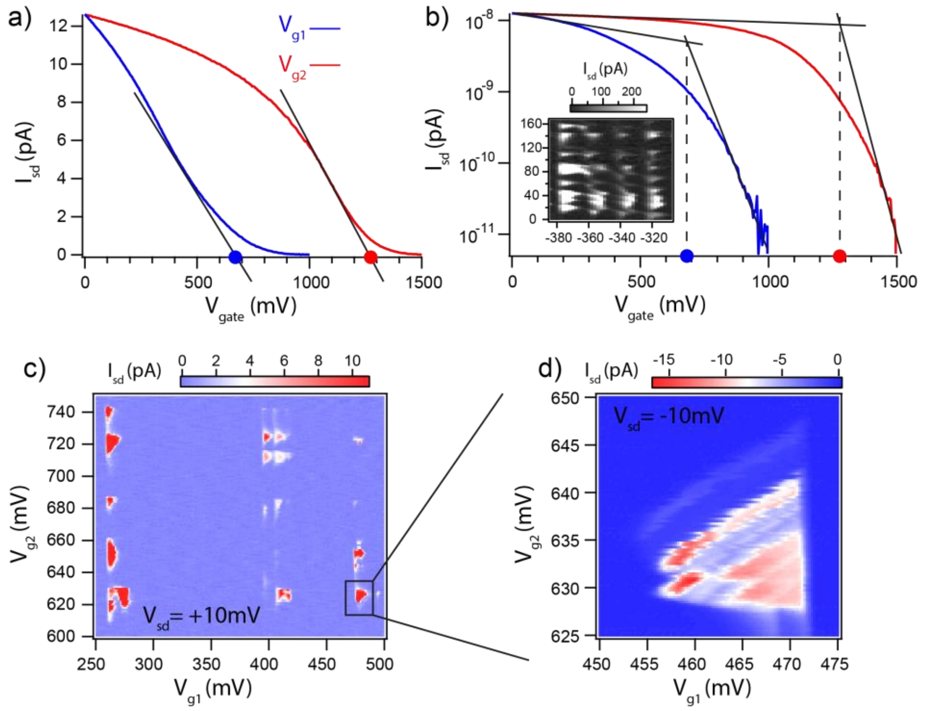

Supplementary Figure 3: ***Extracting the hole occupancy****: a) and b) Source-drain current I_{sd} as a function of gate voltages V_{g1} (in blue) and V_{g2} (in red) at room temperature with V_{sd} = 5 mV and the other gate fixed at 0mV. Superimposed black lines indicate how threshold voltages are estimated. Blue and red dots highlight the values of threshold found for gate 1 and gate 2, respectively. Inset of b): I_{sd} (V_{g1}, V_{g2}) for V_{sd} =10mV. V_{g1} is in along the x-axis while V_{g2} is along y-axis. b) I_{sd} (V_{g1}, V_{g2}) at V_{sd} =10mV containing the EDSR working point of Fig 2a). c) Zoom in c) at V_{sd} =-10mV. The EDSR spectrum presented in Fig.2 in the main text has been recorded at the basis of this bias triangle. Note that this measurement has been taken with a MW signal applied on V_{g1}.*

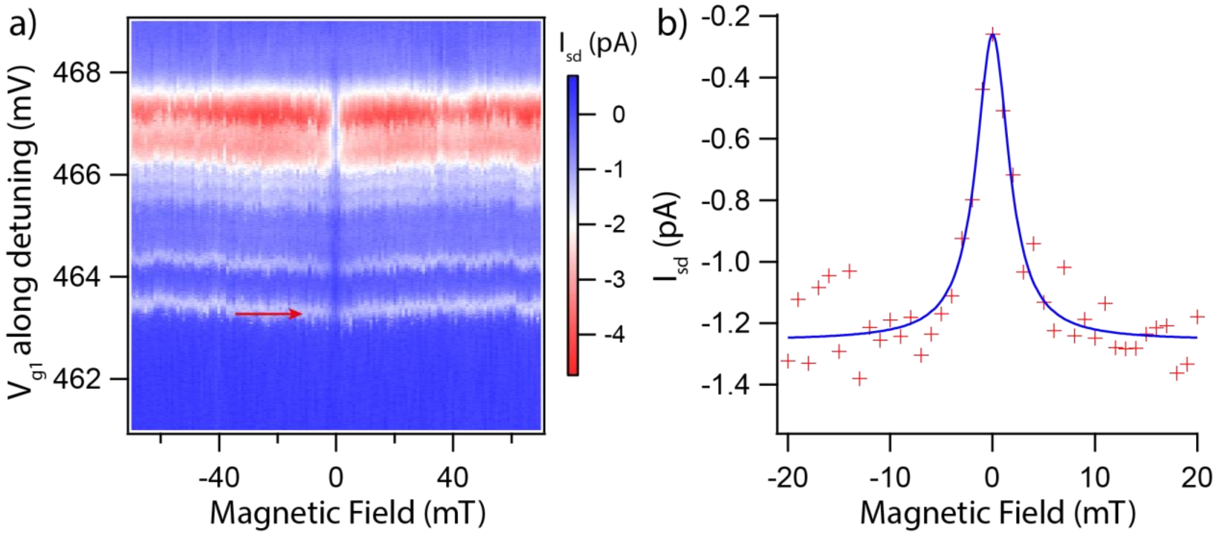

Supplementary Figure 4. ***Pauli spin Blockade****: a) Source-drain current as a function of magnetic field, B, and detuning $\Delta\varepsilon = \varepsilon_1 - \varepsilon_2$ between the electrochemical potentials ε_1 and ε_2 of the two quantum dots (source-drain bias V_{sd} = −10 mV). Detuning is realized through a gate voltage sweep in the (V_{g1},V_{g2}) plane cutting through the base of the current triangle (where $\Delta\varepsilon = 0$) and going through its apex (where $\Delta\varepsilon = eV_{sd}$). Current through the double dot reveals a dip at B = 0 which we*

interpret as due to the onset of spin-orbit mediated spin mixing. b) Horizontal cut at the red arrow in b) (red crosses) and corresponding Lorentzian fit (blue curve).

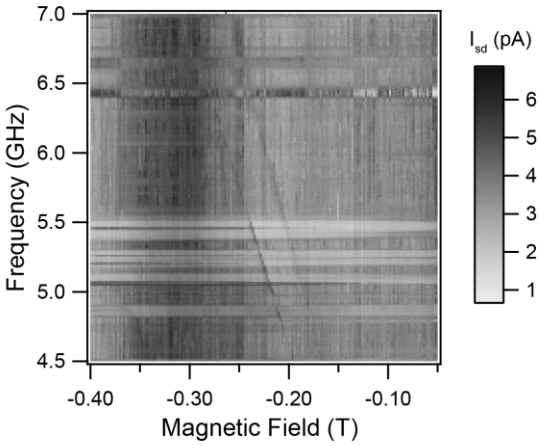

Supplementary Figure 5: ***EDSR****: Current I_{sd} as a function of magnetic field and MW frequency at the same parity-equivalent regime than Fig.2a) but for another frequency range.*

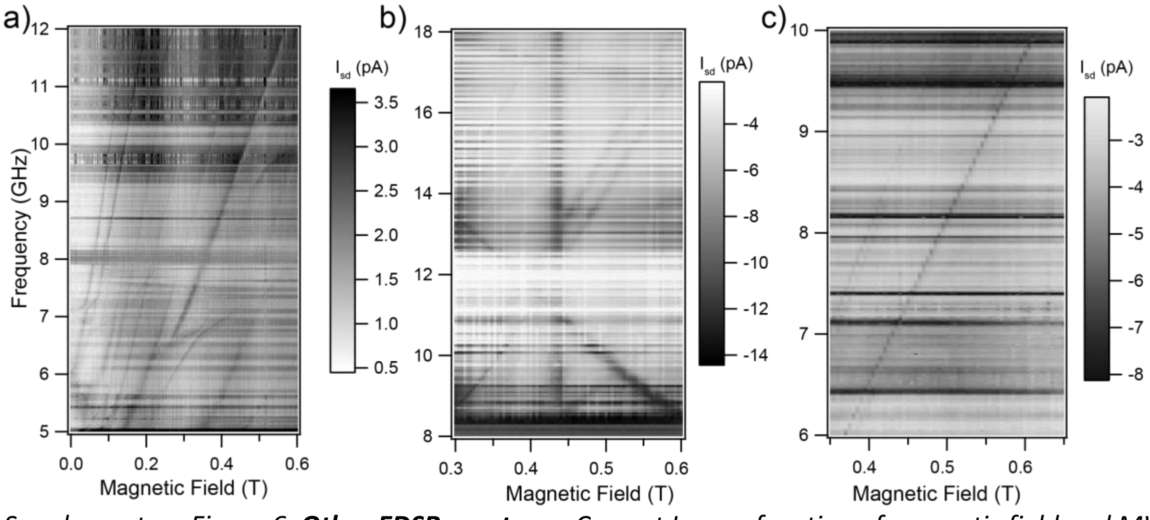

Supplementary Figure 6: ***Other EDSR spectrum****: Current I_{sd} as a function of magnetic field and MW frequency for different Pauli blocked charge configurations of the double quantum dot. The EDSR resonance on which we performed the controlled spin manipulation experiments discussed in the main text is in a). The middle panel corresponds to another charge configuration of the same device. The rightmost panel has been obtained on a second device (the same from which we obtained the data of Fig. 2a, but different working point).*

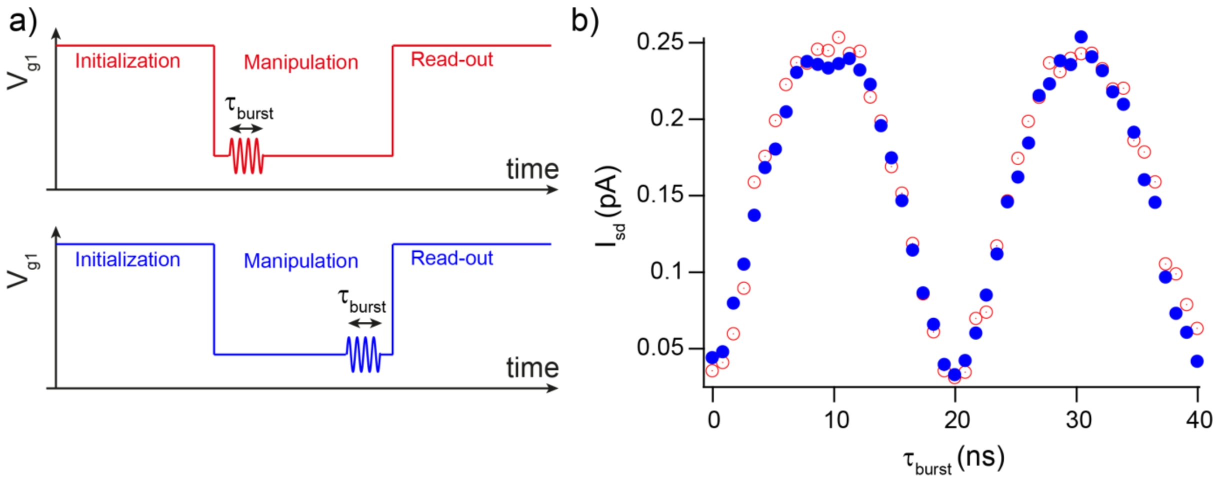

Supplementary Figure 7: ***Spin relaxation:*** *a) Illustration of the manipulation schemes to identify a possible spin relaxation during the manipulation time. b) Rabi oscillations obtained with the manipulation schemes in a).*

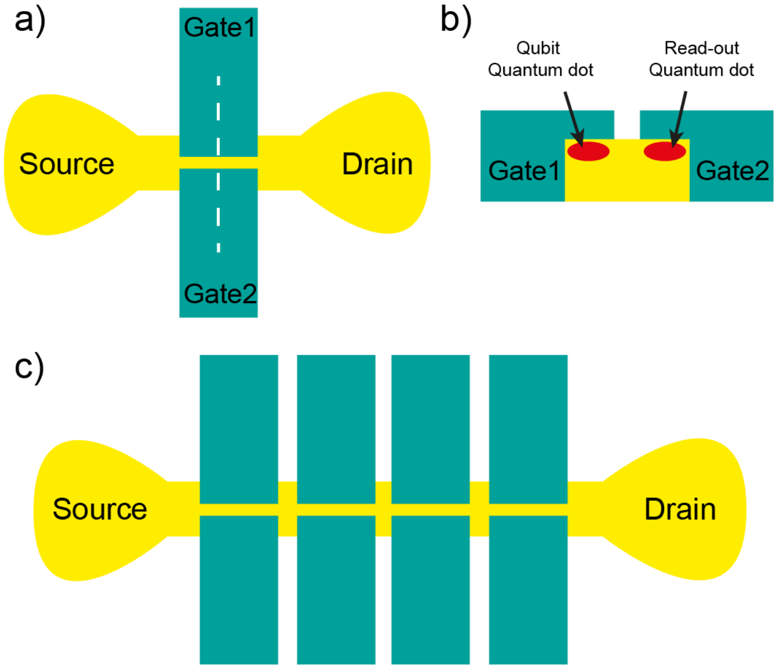

Supplementary Figure 8: ***Face to face geometries:*** *a) Schematic view of a face-to-face device with 2 gates in front of each other. b) Cut across the nanowire along the white dashed line in a). In the face-to-face geometry, two corner quantum dots will be implemented. c) Schematic view of a linear array of face-to-face devices.*

Supplementary Notes

Supplementary Note 1 : Device Fabrication

Experiments are carried out on silicon nanowire field-effect transistors (NW-FETs) fabricated on a 300mm Silicon-On-Insulator (SOI) processing line [1]. Simplified key steps of fabrication are presented in Supplementary Figure 1. First, a silicon nanowire is etched from a SOI wafer with a 10-nm-thick, undoped silicon device layer (see Supplementary Figure 1-a). The nanowire channel is oriented along the [110] direction. Initially defined by deep ultra-violet (DUV) lithography, its width W is trimmed down to about 15 nm by a controlled oxidation and etching process. Two parallel top-gates, ~35-nm wide and with a ~30 nm spacing between them, are successively patterned by means of a combined DUV and e-beam lithography (Supplementary Figure 1-b). The latter enables us to achieve the necessary small spacing between the gates. The gate stack consists of a thin (~5 nm) TiN layer followed by a much thicker (~50 nm) polysilicon layer. Gate electrical isolation is ensured by a dielectric stack consisting of a SiO_2 layer of 7 nm and an Hf-based high-k dielectric layer of 2 nm. Insulating SiN spacers are deposited all around the gates (Supplementary Figure 1c). Their width is deliberately large in order to fully cover the nanowire channel between the two gates and protect it from the successive ion implantation process (Supplementary Figure 1-d), which is required for low resistance ohmic contacts to the nanowire channel. For these p-type devices we use boron ion implantation. Wide spacers also limit boron diffusion from the heavily implanted contact regions into the channel. Dopants are activated by spike annealing followed by self-aligned silicidation. Devices are finalized with a standard microelectronics back-end of line process. At the end, all device fabrication is based on standard processes of our CMOS line, except for the e-beam lithography. We note that gate pitches as small as the one used here, i.e. well below the diffraction limit of DUV (about 190 nm), could as well be obtained with DUV through multiple patterning combined with high-precision realignment [2].

Supplementary Note 2: Hole double QD by accumulation

Supplementary Figure 2 shows a typical double quantum dot transport feature consisting of two closely spaced current triangles [3]. This data was taken at a cryostat temperature T = 10 mK, and for a bias voltage V_{sd} = 5 mV. Inside each triangle, the electrochemical potentials $\varepsilon1$ and $\varepsilon2$ of quantum dots QD1 and QD2 are inside the bias energy window $eV_{sd} = \mu1 - \mu2$. The slopes of the triangle edges (upper edge almost horizontal and left edge almost vertical) indicate a small capacitive cross-talk, i.e. gate 1 (2) has a strong effect on $\varepsilon1$ ($\varepsilon2$) and a weak effect on $\varepsilon2$ ($\varepsilon1$). This behavior is not surprising when we consider that each gate is tightly wrapped around the nanowire shielding the underlying channel from the electric field produced by the other gate. Due to this small capacitive cross-talk, the application of a MW tone on one gate will most likely act on the QD accumulated just below. However since the exact mechanism of spin resonance is not experimentally proven, we cannot exclude that side–ways motion of the hole could be the origin of EDSR.

Supplementary Figures 3-a) and b) present room temperature measurements of current I_{sd} as a function of V_{g1} (in blue) and V_{g2} (in red), with the current plotted in linear and log scale, respectively. In each case, while one gate is swept, the other is kept fixed at zero voltage. The superimposed black lines show how we extract the threshold voltage V_{th} for both gates at room temperature (the values of V_{th} are pinpointed by a blue and a red dot for gate 1 and gate 2, respectively). At low temperature, current transport is fully blocked for V_{g1} or V_{g2} close to the respective threshold voltages. As a consequence, the few hole regime cannot be accessed, and the number of confined holes cannot be precisely counted. To estimate the number of holes in each quantum dot at the charge configuration

for which we perform EDSR, we assume that the first hole enters a QD at V_{th}. This assumption is rather crude (V_{th} is determined at room temperature and with limited accuracy), and an uncertainty of ±100mV seems reasonable based on our experience. As a result, we estimate V_{th1} = 700 ± 100 mV and V_{th2} = 1300 ± 100mV. The next step is to evaluate the gate voltage variation ΔV_g associated with the addition of a single hole. The inset of b) shows a current map $I_{sd}(V_{g1}, V_{g2})$ in the many hole regime, i.e. at relatively low gate voltages. Here, the typical gate voltage between two charge states is ΔV_g ~20 mV. Supplementary Figure 3-c) presents an $I_{sd}(V_{g1}, V_{g2})$ map containing the charge state where we performed EDSR, which is shown in Fig.2 a). In this regime, close to channel pinch-off, not all the current triangles are visible due to the suppressed coupling. A zoom on the EDSR working point, centered at V_{g1} ~ 465 mV and V_{g2} ~ 635 mV, is shown in Supplementary Figure 3-d). Assuming a constant addition energy, we estimate the number of holes in each dot to be: $N_{QD1} = \frac{700\ mV - 465\ mV}{20\ mV} = 12 \pm 5$ and $N_{QD2} = \frac{1300\ mV - 635\ mV}{20\ mV} = 33 \pm 5$.

Supplementary Note 3: Signatures of Pauli Spin Blockade in the presence of spin-orbit coupling

To identify "parity-equivalent" (1,1)→(0,2) regime, we focused on the behavior of the source-drain current as a function of magnetic-field. Depending on the dominant mechanism for spin mixing, the source-drain current can exhibit either a peak either a dip at zero magnetic field. In the following we discuss the different spin mixing mechanisms and their influence on the leakage current characteristic. Finally as a representative example we show in Supplementary Figure 4 the magnetic field dependence of the source-drain current in the parity-equivalent (1,1)→(0,2) regime where we observed the EDSR spectrum displayed in Fig.2a in the main text.

Pauli spin blockade can have different manifestations depending on the dominant mechanism for spin mixing. When the latter is due to hyperfine coupling, a leakage current is observed at zero magnetic field, B, due to nuclear-field induced mixing between the unblocked spin-singlet and the blocked spin-triplet states [4]. This spin mixing mechanism is suppressed by a relatively small B, exceeding the typical average magnitude of the nuclear magnetic field (typically a few mT). This causes the recovery of spin blockade. An alternative mechanism for spin relaxation is spin-flip cotunneling, whereby a second-order tunneling process exchanging an electron with the source reservoir can result in an effective spin flip [5]. This second relaxation channel is also suppressed by an applied B, yet on a field scale set by the electronic temperature in the source reservoir ($g\ \mu_B\ B \sim k_B T_e$), which can be of the order of a few hundred mT in silicon [6]. A third mechanism for spin mixing is spin-orbit coupling. Contrary to the previous cases, this mechanism is inactive at B = 0 due to time-reversal symmetry, but it can become dominant at finite B. As a result, a current dip at B = 0 is expected [7], as indeed observed in double quantum dots formed within low band-gap semiconductors such as InAs [8]. A similar phenomenology is found in holes for silicon [9] and also in the hole double quantum dots studied here. A representative example is shown in Supplementary Figure 4-a). (It corresponds to the same parity-equivalent (1,1)→(0,2) regime where we observe the EDSR resonances displayed in Fig. 2a of the main text.) The source-drain current is plotted as a function of B and detuning $\Delta\varepsilon$ between the quantum dot levels. Current is suppressed around B = 0. A horizontal cut at zero detuning (Supplementary Figure 4-b)) exhibits a clear current dip (note that current is negative due to a negative source-drain bias voltage). Following the transport model developed in [7] the dip can be fitted to a Lorentzian function. In this

regime, the leakage current at finite field is given by the relaxation rate between the (1,1) states. From our fit we extract here a relaxation rate of Γ_{relax}=1.75MHz.

Supplementary Note 4: Electrically driven spin resonance

As mentioned in the main text, recent experiments have revealed the presence of a sizeable spin-orbit coupling in silicon hole quantum dots [9, 10]. A spin-orbit length of l_{SO}~100 nm was extracted in [9]. Even if some caution is required in the interpretation of this finding, this result implies an opportunity for a spin-orbit mediated EDSR. In Ref. [10], the possibility for EDSR based on g-tensor modulation resonance was demonstrated. At this point, the exact origin of the EDSR signal measured here is not fully clear. Further systematic studies are required to address this question (g-factor anisotropy and gate dependence, magnetic-field angle dependence of spin blockade and Rabi oscillations). While certainly very interesting from a fundamental viewpoint, these studies are beyond the scope of the present work, whose focus is on the use of industry-standard CMOS technology for qubit applications. First we present in Supplementary Figure 5 a complementary measurement of the EDSR spectrum displayed in Figure 2-a) of the main text. Eventually in Supplementary Figure 6 we present a few examples of EDSR-induced current resonances for different parity-equivalent (1,1)→(0,2) configurations.

Supplementary Note 5: Spin relaxation

To identify a possible spin relaxation during the manipulation time, we compare Rabi oscillations measured with a MW burst towards the end of the manipulation time, as described in the main text, with those measured with a MW burst at the beginning of the manipulation time. The results, which we show in Supplementary Figure 7-b) (with the corresponding manipulation schemes in Supplementary Figure 7-a)), reveal no appreciable difference, implying no significant relaxation during the manipulation time used in our experiment. This finding is an indication that the characteristic time for spin relaxation, T1, is much larger than the manipulation time (175 ns).

Supplementary Note 6: Prospects for scalable qubit geometries

The first requirement for the development of scalable spin qubit devices is a single-shot readout. The approach followed so far relies on fast measurements of a charge-sensitive device (quantum point contact or a single-electron transistor) lying close to the qubit. The fastest readout is achieved through rf-reflectometry, usually performed with the source contact of the charge sensitive device connected to a LC resonator. Spin detection relies on a spin-to-charge conversion based on either energy filtering (via electron tunneling into a Fermi reservoir) or spin blockade (via electron tunneling to an adjacent quantum dot).

A face-to-face, double-gate transistor of the type used in earlier works [11] and schematically shown in Supplementary Figure 8-a) and b) could provide a minimal device for the implementation of single-shot readout. The spin qubit, confined below gate 1 could be measured by means of a second 'read-out' quantum dot confined under gate 2. In principle, this may simply rely on spin-dependent tunneling of the qubit electron into the readout quantum dot and rf gate reflectometry [12]. The face-to-face gate geometry would as well be suitable for the implementation of linear qubit arrays relying on nearest-neighbor coupling (see Fig.S6-c)). Basic elements such as two-qubit gates (e.g; CNOT) and eventually quantum-error correction could be implemented in this readily accessible linear geometry.

In the prospect of developing surface-code architectures, two-dimensional qubit geometries would be required, which represents a major technical step. At present, different possible development routes can be envisioned, either uniquely based on direct nearest-neighbor exchange, or relying on longer (>100 nm) qubit coupling schemes. In the latter case (which would have the advantage of relaxing the constraint of a very dense qubit "packaging"), the long range coupling could be established through electron shuffling or, in the case of hole-based qubits, through electric-dipole coupling mediated by a microwave resonator or more simply by a floating gate.

Regardless of the scaling approach, we believe SOI nanowire technology offers some interesting advantages. Charge confinement is partly realized by controlled etching of the silicon device layer, which enables reducing the number of gates for each qubit, see for example the realization of a quadruple quantum dots [13]. SOI technology offers valuable opportunities for exploiting integration in the third (out-of-plane) direction as well as for co-integrating classical CMOS electronics together with the qubits layer.

Supplementary References

[1] Barraud, S. *et al.* Performance of omega-shaped-gate silicon nanowire MOSFET with diameter down to 8 nm. *IEEE Electron Device Lett.* **33,** 1526–1528 (2012).

[2] Natarajan, S. *et al.* A 14nm Logic Technology Featuring 2 nd -Generation FinFET Interconnects, Self-Aligned Double Patterning and a 0 . 0588 µm2 SRAM cell size. *Electron Devices Meet. (IEDM), 2014 IEEE Int.* 71–73 (2014).

[3] Wiel, W. G. Van Der *et al.* Electron transport through double quantum dots. *Rev. Mod. Phys.* **75,** 1–22 (2003).

[4] Koppens, F. H. L., Folk, J. A., Elzerman, J. M. & Hanson, R. Control and Detection of Singlet- Triplet Mixing in a Random Nuclear Field. *Science.* **309,** 1346–1350 (2005).

[5] Qassemi, F., Coish, W. A. & Wilhelm, F. K. Stationary and Transient Leakage Current in the Pauli Spin Blockade. *Phys. Rev. Lett.* **102,** 176806 (2009).

[6] Lai, N. S. *et al.* Pauli spin blockade in a highly tunable silicon double quantum dot. *Sci. Rep.* **1,** 110 (2011).

[7] Danon, J. & Nazarov, Y. V. Pauli spin blockade in the presence of strong spin-orbit coupling. *Phys. Rev. B* **80,** 041301 (2009).

[8] Nadj-Perge, S. *et al.* Disentangling the effects of spin-orbit and hyperfine interactions on spin blockade. *Phys. Rev. B* **81,** 201305 (2010).

[9] Li, R., Hudson, F. E., Dzurak, A. S. & Hamilton, A. R. Pauli Spin Blockade of Heavy Holes in a Silicon Double Quantum Dot. *Nano Lett.* **15,** 7314–8 (2015).

[10] Voisin, B. *et al.* Electrical Control of g-Factor in a Few-Hole Silicon Nanowire MOSFET. *Nano Lett.* **16,** 88–92 (2016).

[11] Roche, B. *et al.* Detection of a large valley-orbit splitting in silicon with two-donor spectroscopy. *Phys. Rev. Lett.* **108,** 206812 (2012).

[12] Betz, A. C. *et al.* Dispersively Detected Pauli Spin-Blockade in a Silicon Nanowire Field-Effect Transistor. *Nano Lett.* **15,** 4622–7 (2015).

[13] Betz, A. C. *et al.* Reconfigurable quadruple quantum dots in a silicon nanowire transistor. *Appl. Phys. Lett.* **108,** 203108 (2016).
